# The Limbic System in Co-Occurring Substance Use and Anxiety Disorders: A Narrative Review Using the RDoC Framework

**DOI:** 10.3390/brainsci14121285

**Published:** 2024-12-21

**Authors:** Esther R.-H. Lin, Faith N. Veenker, Peter Manza, Michele-Vera Yonga, Sarah Abey, Gene-Jack Wang, Nora D. Volkow

**Affiliations:** Laboratory of Neuroimaging, National Institute on Alcohol Abuse and Alcoholism, National Institutes of Health, Bethesda, MD 20892, USA; esther.lin@nih.gov (E.R.-H.L.); faith.veenker@nih.gov (F.N.V.); peter.manza@nih.gov (P.M.); michele-vera.yonga@nih.gov (M.-V.Y.); sarah.abey@nih.gov (S.A.); nora.volkow@nih.gov (N.D.V.)

**Keywords:** SUDs, substance use, addiction, anxiety, neuroimaging, fMRI, limbic system, RDoC

## Abstract

Substance use disorders (SUDs) and anxiety disorders (ADs) are highly comorbid, a co-occurrence linked to worse clinical outcomes than either condition alone. While the neurobiological mechanisms involved in SUDs and anxiety disorders are intensively studied separately, the mechanisms underlying their comorbidity remain an emerging area of interest. This narrative review explores the neurobiological processes underlying this comorbidity, using the Research Domain Criteria (RDoC) framework to map disruptions in positive valence, negative valence, and cognitive systems across the three stages of the addiction cycle: binge/intoxication, withdrawal/negative affect, and preoccupation/anticipation. Anxiety and substance use play a reciprocal role at each stage of addiction, marked by significant psychosocial impairment and dysregulation in the brain. A more thorough understanding of the neural underpinnings involved in comorbid SUDs and anxiety disorders will contribute to more tailored and effective therapeutic interventions and assessments.

## 1. Introduction

In the United States, substance use disorders (SUDs) are highly prevalent and costly. Drug and alcohol use contributed to nearly 11 million premature deaths in 2019 alone [1]. Effective treatment of SUDs is particularly challenging due to the significant comorbidity between SUDs and other mental health conditions. Among concomitant mental health diagnoses, mood and anxiety disorders have strong reciprocal relationships with SUDs [2,3]. For example, drug withdrawal can result in well-characterized autonomic and somatic symptoms that classically accompany anxiety [4,5]. Consequently, psychological symptoms accompanying withdrawal, like anxiety, may be mistaken as part of withdrawal rather than an underlying separate mood disorder that requires careful treatment [6]. In addition, aversive affective withdrawal symptoms, such as anxiety, can contribute to the escalation of compulsive drug use, maintenance of use, and relapse after periods of abstinence, i.e., negative reinforcement of drug addiction to alleviate negative emotional states [7,8,9].

**Figure 1:**
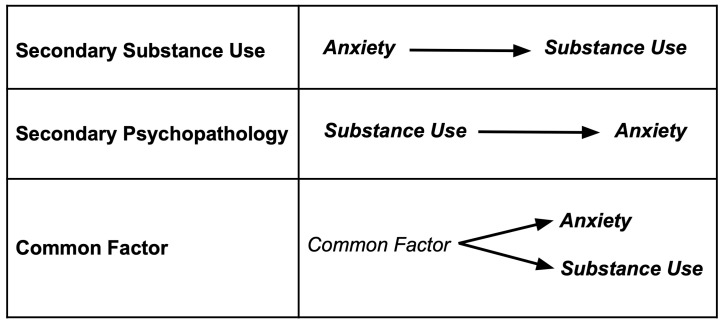
Three possible explanatory models of the relationship between anxiety disorders and substance use disorders: secondary substance use, secondary psychopathology, and common factor. Arrows indicate direction of causality.

Three explanatory models about the potential causal impacts of chronic anxiety on substance use (and vice versa) are discussed in this review (Figure 1). These three models are not mutually exclusive, as we will present throughout the paper [10]. First, having a psychiatric disorder increases susceptibility to addictive behaviors because certain substances may temporarily reduce symptoms of mental conditions and act as a negative reinforcer [11]. This is termed the secondary substance use model and embraces the self-medication hypothesis, in which the constant use of substances to remedy the negative affect states of psychiatric conditions leads to an SUD diagnosis [12]. Social anxiety disorder (SAD) has been identified as an example of such a relationship, but for other anxiety disorders, the temporality with SUD development remains unclear [13]. Second, the secondary psychopathology model proposes that SUDs may promote vulnerability to anxiety disorders due to the consequences of chronic drug use or related withdrawal symptoms [12,14]. A study using National Epidemiological Survey on Alcohol and Related Conditions (NESARCs) data found a higher likelihood of individuals having alcohol use disorder (AUD) as their primary diagnosis among patients with comorbid panic disorder or generalized anxiety disorder [13]. Third, the common factor model suggests that shared genetic and environmental factors (e.g., traumatic or stressful life experiences) may mediate the relationship between SUDs and anxiety rather than reflect a direct causal association [12,15,16]. The self-medication hypothesis in the secondary substance use model appears to be the most commonly used explanation for comorbid SUDs and anxiety. The literature has demonstrated empirical evidence that supports this hypothesis in comorbid social phobia and AUD, as well as comorbid post-traumatic stress disorder (PTSD) and SUDs. However, since various anxiety disorders interact differently with different SUDs, there are potentially other mechanisms that explain their comorbidity [12]. Neurobiological study of the pathogenesis and evolution of comorbid SUDs and anxiety disorders is crucial to investigate these models, advance treatment, and improve outcomes.

One approach for the purpose of investigating psychiatric comorbidities is to use the Research Domain Criteria (RDoC), a multi-system framework that maps domains of neurobehavioral functioning to mental disorders. Since its conception in 2008, RDoC has been established as a valid framework that is particularly valuable when studying comorbidities due to its multidimensional approach (e.g., integrating neurobiological and psychosocial factors) and focus on common underlying constructs of multiple disorders (e.g., disruption to positive valence systems as a core feature of both SUDs and anxiety) [17,18]. Prior studies have successfully applied the RDoC framework to produce clinically relevant insights for depressive, personality, and psychotic disorders [19,20,21]. The RDoC framework is a step toward precision psychiatry [22], with one of its greatest strengths being its ability to guide “attempts to associate underlying psychological constructs of cognition and emotion with specific neural circuitry” [23]. Though the RDoC is a strong framework, one notable weakness is the lack of recognition of the dynamic nature of psychiatric illness onset and progression [23]. We aim to address this by discussing the potential impact of early life stress on one’s probability to engage in substance use through the reciprocal and causal effect of anxiety and the SUD cycle. Current neuroimaging studies provide valuable insights about the neural underpinnings of anxiety or SUDs, and the RDoC framework provides a unified way to integrate the two veins of research to identify novel transdiagnostic biomarkers for treating their comorbid presentations. Here we examine SUD-AD comorbidity through this lens, conducting a narrative review of studies that highlight limbic system neuroadaptations associated with these disorders.

## 2. Methodology

Between the months of August 2024 and October 2024, we searched the database PubMed and the search engine Google Scholar using the following terms: “Anxiety and SUD OR Addiction OR SUD OR Anxiety OR Neuroimaging or RDoC.” Given the broad scope of the literature on anxiety and SUDs, we restricted our search to studies from 1990 to 2024. Additional articles were identified via recursive reference searching and previous knowledge. For the purposes of this review, we included studies that looked at anxiety disorder diagnoses from multiple iterations of the DSM (i.e., panic disorder, generalized anxiety disorder, social anxiety disorder). We also included studies that describe state and trait anxiety in comorbid SUDs. Research articles published prior to 1990 were excluded. Given that PTSD has a distinct classification from anxiety disorders in the DSM-V, where possible, we separately highlight findings related to PTSD. We also excluded studies on obsessive-compulsive disorders, which are categorized by DSM-V as separate from anxiety disorders, despite having overlapping symptoms. To allow for a diverse understanding of the neuroadaptations present in comorbid SUD-AD, the exclusion criteria were kept broad.

## 3. Dimensional Frameworks for Understanding Comorbidity

The RDoC provides a transdiagnostic, systematic framework to characterize shared neurobiological processes underlying SUDs and ADs across domains. The RDoC matrix covers six domains: negative valence, positive valence, cognitive, social processes, arousal/regulatory, and sensorimotor systems. The Alcohol and Addiction Research Domain Criteria (AARDoC) further delineates the major underlying domains of functions relevant to SUDs: negative emotionality (mapping onto the negative valence system), incentive salience (mapping onto the positive valence system), and executive function (mapping onto the cognitive system). We note that the social processing, arousal, and sensorimotor systems are also relevant for SUDs but here we focus on three relevant domains that correspond with the three stages of the addiction cycle proposed by Litten and colleagues [24].

Current neuroscience-based models conceptualize SUDs as a three-stage cyclical process that intensifies with continuing drug use. These stages include (1) binge and intoxication, (2) withdrawal and negative affect, and (3) preoccupation and craving [25]. The three stages in the addiction cycle represent three functional psychobiological constructs: incentive salience or pathological habits, reward deficit or stress surfeit, and executive dysfunction. Anxiety can be conceptualized as either state anxiety (a transient reaction to a stressor) or trait anxiety (an enduring attribute) [26]. ADs, such as generalized anxiety disorder (GAD) involve high levels of trait anxiety, but people with ADs may also be more reactive to transient stressors as well [27]. Changes in anxiety levels, marked by a state of distress and arousal, are relevant to all three stages of SUDs. Given significant overlaps between SUDs and anxiety in various domains, especially negative valence and cognitive systems, this psychobiological framework is of particular relevance to the SUD-AD comorbid condition. Core dysfunctions of SUDs and ADs and their manifestations were investigated in the context of the RDoC matrices for negative valence, positive valence, and cognitive systems for each disorder.

Below, we summarize the basic neurobiology underlying each process, followed by a narrative review of the relevant neuroimaging data.

### 3.1. Positive Valence Systems—Binge/Intoxication

#### 3.1.1. Neurobiology

Individuals with anxiety often exhibit diminished sensitivity in positive valence systems, including the motivation to obtain rewards [28,29,30]. This is evidenced by decreased activation of the striatum to reward feedback [31]. Blunted reward processing to natural rewards may prime people with anxiety disorders to seek out other experiences that increase reward signaling, such as substance use [32]. During the binge and intoxication phase, consuming drugs increases activity related to positive valence systems, such as reward seeking and habit learning [33]. The mesolimbic and the mesocortical dopamine systems are highly involved in this drug-induced reward processing. The mesolimbic system includes neurons that originate in the ventral tegmental area (VTA) and project upwards to the ventral striatum (VS), including the nucleus accumbens. Drug consumption induces rapid ‘bursts’ of dopamine release in the VS (also known as phasic dopamine release) [34], which activate dopamine D1 receptors and are associated with the rewarding experience of feeling “high” or euphoric [35,36]. The adjacent nigrostriatal dopamine system has been classically associated with motor control, but it also plays a role in reward processing. This pathway originates in the substantia nigra and projects upward to the dorsal striatum (DS). Like in the VS, drug consumption also increases dopamine release in the DS, though to a lesser extent than in the other dopamine systems [35,36].

With long-term drug use, an individual’s reward processing distorts to disproportionately favor drug consumption over other rewards. This reflects, in part, long-term potentiation (LTP), which is one of the mechanisms driving neuroplasticity in the brain triggered by drug-induced signaling [37]. These neuroplastic changes drive incentive salience, i.e., heightened or focused attention toward cues linked to rewarding behaviors [38]. Similar to the mesolimbic system, the mesocortical system also originates in the VTA and includes neuronal projections to the medial prefrontal cortex (mPFC). Release of dopamine into the mPFC cues incentive salience, which increases motivation for further drug consumption [25,36]. This is further evidenced by research that has found that as an individual starts to develop drug-cue associations, phasic dopamine bursts also occur in response to drug cues [39]. Over time, drugs become a preferred specific stimulus, and reward circuitry activation in response to non-specific, non-drug stimuli (e.g., social interaction, daily activities) gradually diminishes. This diminished reward processing to “natural” rewards can provoke anxiety, as individuals face chronic anhedonia and are provoked to further drug consumption [40].

#### 3.1.2. Neuroimaging Findings: Anxiety → Binge/Intoxication

Stress-induced early life programming can sensitize reward circuitry and make someone more prone to engaging in substance use [41]. Stress in early life is associated with higher levels of anxiety expression [42], and is a risk factor for early drug initiation [43] and SUDs [44]. A recent review found that anxiety sensitivity is associated with both PTSD and SUDs, suggesting it may play a pivotal role in their co-occurrence [45]. Events that induce PTSD may also prompt increased trait anxiety [46] and propensity toward substance use [47]. Luckily, evidence shows that several types of treatments (trauma-focused, non-trauma-focused, and manualized SUDs) can be effective interventions for co-occurring PTSD and SUDs [48]. There is neurobiological evidence for these ideas; neuroimaging studies find acute intoxication could potentially counteract anxiety-related neural dysregulation. Anxiety is associated with dampened or dysregulated activity in the VS [49,50], so during intoxication, the supraphysiological bursts of VS dopamine and heightened VS activity may overcome these deficits [25,51,52]. As people consume drugs more frequently, drug cues alone can also prompt increased VS activity [53]. Further, many anxiety disorders are consistently associated with increased activity in fear-related limbic regions including the amygdala [54], particularly in response to relevant anxiety cues [55]. Similar to how acute intoxication can increase VS activity to potentially counteract dysregulated reward-related activity in people with anxiety, some studies have found that intoxication, particularly with alcohol, can acutely decrease amygdala reactivity [56,57].

#### 3.1.3. Neuroimaging Findings: Binge/Intoxication → Anxiety

While drugs may temporarily relieve feelings of anxiety, chronic self-medication leads to maladaptive neuroplastic changes that decrease the value of drugs. For example, with long-term substance use, stimulant drugs do not induce an increase in dopamine signaling to the same degree as healthy controls [52]. Simultaneously, changes in the nigrostriatal system may support habit formation [58], which may be why drug cues alone are sufficient to induce dopamine increases in dorsal striatum in SUDs [59]. Intriguingly, a case study found that damage to the dorsal striatum completely reversed addictive behavior [60]. Over time, a paradoxical brain response develops; dopamine responses to drug consumption decrease while incentive salience increases [25,61]. This mismatch results in increased “wanting” and decreased “liking” [61].

Impaired reward prediction error (RPE) learning underlies the drive to seek rewards in SUDs [62] and can lead to increased negative emotions and feelings of anxiety, as the experience of drug consumption does not match the anticipated response. Disrupted dopamine RPE signal is associated with neuronal plasticity alterations in the striatum, frontal cortex, and amygdala [63]. Behaviorally, prediction errors are also implicated in anxiety disorders as wrong expectations of danger can trigger excessive fear levels and motivate avoidance behaviors [64]. Clinical studies showed that prediction error signaling is disrupted in patients with GAD completing a passive avoidance task and associated their performance with the ventromedial prefrontal cortex (vmPFC) and ventral striatum regions. Individuals with SAD also show similar trends but with elevated dmPFC activation correlating to prediction errors, and reduced dmPFC-ventral pallidum connectivity [65].

### 3.2. Negative Valence Systems—Withdrawal/Negative Affect

#### 3.2.1. Neurobiology

Withdrawal/negative affect follows the binge/intoxication stage of the addiction cycle and includes core components of anxiety disorders. During withdrawal, dopamine transmission decreases in mesolimbic circuits, which is associated with motivational deficits and decreased mood [37]. Due to neuroadaptations associated with long-term drug abuse, nothing relieves this negative affect except drug consumption. This is because drugs preferentially act on neural reward circuitry that has been altered from chronic drug use [66]. As dependence and withdrawal develops, the brain’s anti-reward system is activated and negative affective symptoms emerge including anxiety, irritability, and hyperkatifeia [67]. While different drugs can elicit different withdrawal symptoms, negative affect is a universal and motivational element of withdrawal [68]. These negative emotional states spur further drug use to avoid discomfort, i.e., negative reinforcement [69]. Individuals with a history of anxiety experience more intense withdrawal symptoms and withdrawal-related discomfort and relapse from substances like methamphetamine, nicotine, or alcohol [70,71], and baseline diagnosis of anxiety disorders like SAD and panic disorder significantly predicted early alcohol relapse [72].

These negative emotional states are driven by increased corticotropin-releasing factor (CRF), noradrenaline, and dynorphin signaling [67]. Injection of CRF antagonists into the central amygdala reduces anxiogenic-like states in rats withdrawn from substances including alcohol, nicotine, and cocaine [73,74,75,76]. Further, noradrenergic antagonists administered into the bed nucleus of the stria terminalis (BNST) reversed opioid-withdrawal-induced place aversion in rodents [77]. Recent evidence suggests changes in endogenous endocannabinoid systems in the extended amygdala are associated with heightened anxiogenic responses in rats with cannabis withdrawal undergoing classical anxiety paradigms like the elevated plus-maze and the light/dark box [78,79,80]. Clinically, classical medications used to treat alcohol and heroin withdrawal include α-adrenergic drugs that inhibit noradrenergic release [81,82]. Together, these findings highlight the importance of stress signaling in the extended amygdala, a component of the limbic system that is linked with anxiety disorders as demonstrated in human imaging studies.

#### 3.2.2. Neuroimaging Findings

While the directionality between anxiety and SUDs specific to the withdrawal stage remains hard to disentangle through neuroimaging research, we present general findings regarding neurobiological overlap between withdrawal and anxiety phenotypes.

The amygdala is heavily implicated in the withdrawal/negative affect stage and increased connectivity in amygdala-related networks is associated with increased emotional reactivity [83,84]. Alterations in connectivity between large-scale brain networks, such as the salience network (SN, including the insula, anterior cingulate, amygdala, and hypothalamus) and the default mode network (DMN, including the medial prefrontal and parietal cortex) are critically involved in both withdrawal and anxiety. SN-DMN connectivity is elevated during withdrawal from substances such as tobacco and alcohol [85,86]. Elevated SN-DMN connectivity is also commonly observed in adolescents and adults with anxiety disorders compared to healthy controls [87,88,89,90], and positively correlates with anxiety severity among both anxiety patients and healthy controls [87,91,92]. While state anxiety is associated with SN-DMN connectivity, specifically in the ventral nodes, recent studies found that trait anxiety is more closely related to the DMN. Individuals with higher trait anxiety exhibit increased resting-state functional connectivity between the DMN and prefrontal areas [26] and reduced intra-DMN connectivity compared to those with low trait anxiety [93].

These connectivity changes are thought to direct attention towards withdrawal-induced physiological sensations, heightening internalizing and anxiety symptoms [94]. For example, both nicotine and alcohol withdrawal [95], self-reported anxiety [96], and irritability [97] have all been linked to elevated amygdala-insula functional connectivity. This may explain why individuals with a history of anxiety experience more intense withdrawal symptoms and withdrawal-related discomfort and relapse from substances like methamphetamine, nicotine, or alcohol [71], as increased amygdala-insula reactivity has been observed in anxiety patients across all ages [98,99,100,101].

As mentioned above, α-adrenergic drugs are effective in treating alcohol withdrawal symptoms, and imaging evidence supports this. Prazosin treatment improves dysregulation in DMN regions including the medial prefrontal cortex (mPFC), which has been associated with severity of alcohol withdrawal symptoms [102] and implicated in anxiety psychopathology [103]. While the SN is involved in processing internal or external stimuli, and mediates the switching between the DMN and the executive control network (ECN), the DMN plays a central role in self-referential processes (evaluating salience or internal and external cues, remembering the past, and planning the future) implicated in affective disorders like anxiety [104] and in SUDs [36]. Dysfunctions in within-network and between-network DMN connectivity are heavily implicated across all anxiety disorders [105], and recent studies have suggested DMN aberrations may underlie anxiety symptoms such as perseverative thoughts in generalized anxiety disorder (GAD) [106,107] and possibly predict anxiety disorders such as GAD and SAD [107,108]. Therefore, among the existing resting-state networks, the DMN may be of particular interest in comorbid SUD-anxiety conditions during the withdrawal stage.

Chronic drug use has opposing effects on DMN engagements, which may contribute to the predominance of anti-reward systems during the withdrawal/negative affect stage [105]. The DMN can be further split into anterior and posterior portions, and reduced anterior-posterior DMN connectivity has been associated with impaired self-awareness [109], and in addicted persons this contributes to uncontrolled drug-taking [110,111]. Increased posterior DMN involvement may underlie ruminatory behaviors, resulting in distress and negative emotional states like anxiety [112,113]. Resting-state studies in individuals addicted to substances like alcohol, heroin, or cannabis found that chronic drug use enhances posterior DMN, and exacerbates the imbalance of anterior-posterior DMN connectivity [36,114,115]. Decreased connectivity between the posterior and anterior DMN may contribute to the inability to inhibit negative emotional states, interfering with abstinence and flexible decision-making which could further exacerbate the anxiety symptoms experienced during withdrawal [25].

Emerging evidence also highlights the importance of the BNST in withdrawal-related anxiety and relapse as a critical node in both the stress and reward circuitries [2]. During withdrawal, the amygdala releases CRF on receptors in the BNST [116], and heightened amygdala-BNST connectivity was observed in people undergoing alcohol withdrawal [86,117]. The BNST is involved in withdrawal-related anxiety and relapse, serving as a critical node in both stress and addiction circuitry with connections to multiple limbic and brainstem regions including the amygdala and VTA. fMRI studies using shock paradigms described increased BNST engagement mediating sustained fear responses, and activity correlated with physiological skin conductance and self-reported anxiety ratings [118,119,120,121].

### 3.3. Cognitive Systems—Preoccupation/Anticipation

#### 3.3.1. Neurobiology

Commonly known as “craving”, the preoccupation/anticipation stage of addiction is characterized by a disruption of executive function and involves the prefrontal cortex. Studies have linked poor executive function (i.e., inhibition, working memory) with early initiation of alcohol, tobacco, and other substances [122,123,124]. Early life stress can exacerbate this vulnerability by inducing alterations in brain functional connectivity in emotion and reward circuits, and these alterations subsequently affect anxiety levels throughout development [125]. These neurobiological disruptions may create a feedback loop that increases vulnerability to substance abuse. Further, acute craving, which is more intense in comorbid AD and SUDs [126], decreases the ability to divert attention away from drug cues [127,128]. Both cue- and stress-induced craving and anxiety are linked to early attrition from alcohol use disorder treatment, and these observations are in part associated with dysregulated hypothalamic-pituitary-adrenal (HPA) axis function [129].

Prior studies found working memory, attentional control, and inhibitory control to be among the most common cognitive domains impacted by anxiety as well as SUDs. Reciprocal glutamatergic connections between the PFC and the dorsal striatum are heavily involved in the flexible control of these behaviors [130]. Preclinical evidence showed that rats withdrawn from substances like cocaine or methamphetamine were unable to develop long-term potentiation and long-term depression in the nucleus accumbens region after stimulating the PFC, suggesting that chronic substance use depresses PFC-accumbens synapses [131,132]. However, treatment using N-acetylcysteine, an agent which acts as a physiological reservoir of neuronal glutamate, can prevent relapse by activating cystine–glutamate exchange in the cortico-accumbens circuitry [133]. This finding is consistent in human studies that reported decreased desire to use drugs in response to cocaine cue reactivity [134].

Persistent prefrontal dopamine D1 receptor signaling is crucial for working memory, a foundational cognitive function [135,136,137]. Nonhuman primate studies showed blocking D1Rs in the PFC impaired learning of novel associations and decreased cognitive flexibility during a working memory task [136,138], and human studies reported that systemic administration of a mixed D1/D2 agonist facilitated working memory while a D2 agonist had no effect [139]. Striatal dopamine D1 versus D2 receptor signaling is also implicated in flexible control of behavior. Animal studies using the probabilistic reversal learning paradigm found that positive feedback learning is modulated by D1R signaling in the ventral striatum while D2R signaling modulates negative feedback learning. Stimulation of D2R in the ventral or dorsolateral striatum promoted explorative choice behavior [140] and reversal learning was impaired by D2R antagonism but not D1R antagonism in the dorsal striatum [141]. Studies have provided evidence that dorsal striatum D1 medium spiny neurons (MSNs) mediate reward/reinforcement and D2-MSNs mediate aversion [142]. Further, rats completing an instrumental task showed that D1R inhibition and D2 activation both promote the expression of flexible responding after development of habitual or compulsive-like behaviors [143].

#### 3.3.2. Neuroimaging Findings: Anxiety → Preoccupation/Anticipation

During the preoccupation and anticipation phase of the addiction cycle, attention to drug cues is heightened [37,144]. Anxiety differentially affects proactive and reactive control, which both dynamically mediate cognitive control. State anxiety induced by the threat of shock impairs proactive control, which suggests that increased state anxiety interferes with the working memory and goal-directed attentional control involved in drug use [145]. Reduced proactive control has been observed in individuals with methamphetamine or cocaine use disorder which may account for the role of poor executive function in relapse vulnerability [146,147]. Neuroimaging studies of cue reactivity have found increased activation related to craving for alcohol, nicotine, or cannabis in cognitive control regions, such as the anterior cingulate cortex (ACC) and vmPFC and limbic regions like the ventral striatum [144,148,149,150]. Alcohol-cue-elicited activation of the ventral striatum correlated with behavioral measures [149] and nicotine craving correlated with left vmPFC and amygdala activation when viewing smoking-related pictures [144]. People with anxiety disorders also exhibit problems with cognitive flexibility [151], and high trait anxiety is linked to low PFC activity during attentional control [152] and dysregulated connectivity between PFC and subcortical regions including the amygdala, basolateral amygdala (BLA), insula, and hippocampus [151,153,154,155,156,157]. In task-based fMRI studies with the stop signal task, amygdala activation positively correlated with trait anxiety, and with the vmPFC during risk taking. Using the same SST task in cocaine-dependent patients [158], vmPFC activation correlated negatively with improved inhibitory control during methylphenidate treatment [159]. Together, these findings suggest that anxiety-related dysregulation in cognitive systems, combined with increased amygdala activation and stress symptoms associated with the preoccupation phase [25], may worsen or reinforce altered cognitive control associated with cue reactivity.

#### 3.3.3. Neuroimaging Findings: Preoccupation/Anticipation → Anxiety

Long-term substance use creates a paradoxical effect in which reward responses are blunted while anticipation heightens [25,61]. During this preoccupation and anticipation phase, many factors promote craving. Neural biomarkers like the insula, hippocampus, and prefrontal cortex are implicated in the craving stage [37]. The insula, particularly its anterior regions, is reciprocally connected to several limbic regions and is responsible for an interoceptive function (integrating autonomic/visceral information with emotion, motivation, and conscious awareness) [160].

SUDs impair interoception [161] and damaged interoceptive awareness is likely one of the contributing factors to why people with SUDs continue substance use [162]. Chronic drug use can lead an individual to associate interoceptive cues with the rewards of drugs to trigger cravings, as the body is reminded of the positive effects of drugs and primes the urge to seek drugs. The imbalance between interoception/exteroception causes an increased focus on external stimuli (i.e., drug cues) and a decreased ability to perceive internal bodily states [97,163]. Neuroimaging studies demonstrated insula activity in response to drug cues in patients addicted to cigarettes, cocaine, and alcohol, and correlated insula activity with self-reported cravings [160,164,165,166,167]. SUD patients with lesions to the insula (or even other regions functionally connected to the insula) can remarkably have a complete remission of their addiction and no longer experience drug cravings or relapse [168,169]. The anterior insula is reciprocally connected to several limbic regions, and cue-reactivity fMRI studies on alcohol-related craving found increased connectivity between the left insula and left dorsomedial PFC but decreased connectivity in a network including the ACC, insula, and hippocampus [170].

The insula, particularly the anterior insula, is also heavily implicated in the pathophysiology of anxiety and anxiety disorders [171,172,173]. Alvarez et al. found that individuals with greater anxiety proneness and lower perceived control showed greater activity in the anterior insula during anticipation of an unpredictable threat [174]. Individuals who are prone to anxiety experience augmented signaling between observed and expected body state, and may use worrying about possible aversive outcomes as a cognitive avoidance response to stressful, unpredictable life events [172,175]. Similarly, addicted individuals with diminished interoceptive abilities may have difficulties regulating internal/external states in response to stressful situations, and turn to further substance use like alcohol as a coping mechanism [176,177,178]. Given the overlap in dysregulated insular activity and interoceptive abilities underlying both SUDs and anxiety disorders, it is likely that anxiety symptoms are amplified or anxiety disorders emerge during the preoccupation/anticipation stage.

## 4. Limitations

Due to the cross-sectional nature of many neuroimaging studies, it is often difficult to determine directionality of causality, i.e., whether the difference in neural activity is a cause or consequence of the studied behavior. Further, literature reviews conducted in a narrative style are inherently prone to author bias. We made a strong effort to mitigate this potential bias by completing a comprehensive search as described in the methodology section. Nonetheless, we acknowledge that this review does not exhaustively cover every possible prior study in this space. Finally, the RDoC framework is continually evolving as neuroscientific research develops, and its application to AD-SUD comorbidity may change accordingly.

## 5. Conclusions and Future Directions

**Figure 2:**
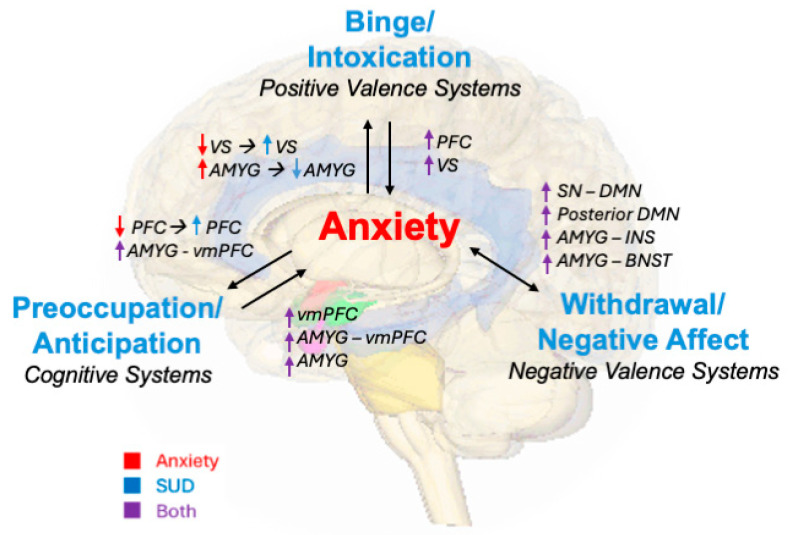
Conceptualizing the comorbidity between addiction neurocircuitry domains and anxiety using task-based and resting-state fMRI neuroimaging findings. Anxiety leading to binge/intoxication (positive valence) is associated with increased VS activity and decreased AMYG activity while binge/intoxication leading to anxiety is associated with increased PFC and VS activity. Bidirectional anxiety and withdrawal/negative affect are associated with increased SN-DMN, AMYG-INS, and AMYG-BNST connectivity, and increased posterior DMN activity. Anxiety leading to preoccupation/anticipation (cognitive) is associated with increased PFC activity and increased AMYG-vmPFC connectivity while preoccupation/anticipation (cognitive) leading to anxiety is associated with increased vmPFC and AMYG activity, and increased AMYG-vmPFC connectivity. Colored arrows depict the associated mental health condition (red = anxiety, blue = SUD, and purple = both). VS = ventral striatum. AMYG = amygdala. PFC = prefrontal cortex. vmPFC = ventromedial prefrontal cortex. INS = insula. BNST = bed nucleus of the stria terminalis. SN = salience network. DMN = default mode network. Limbic brain image is open access, modified from lifesciencedb [179].

Changes in neurobiological circuits underlying ADs and SUDs exacerbate symptoms as individuals cycle through the three stages of addiction. Anxiety can prompt people to consume drugs, which temporarily recruits positive valence systems and increases incentive salience over time. During withdrawal, activity in negative valence systems heightens, and anxiety symptoms increase rapidly. As people enter into the preoccupation and anticipation phase, changes to cue reactivity and interoception are reflected in cognitive systems. The cycle repeats as people become intoxicated again due to lack of cognitive control and increased incentive salience and cue reactivity. As tolerance increases, people binge with increasing drug quantities and neuroplastic changes are reinforced. Each stage of the cycle can worsen preexisting or SUD-induced anxiety. Common findings from task and resting-state fMRI are related to these processes as summarized in Figure 2.

Encouragingly, longitudinal studies of people with SUDs in recovery have found that drug-related functional deficits can be at least partially reversed with abstinence. For example, an investigation of people in recovery from opioid addiction found improvements in nucleus accumbens function in response to reward [180]. An increase in DA transporters after abstinence was observed in methamphetamine users [181], and DA transporter binding in alcohols matched healthy controls after a 4-week period of abstinence [182]. There is also some promising research that anxiety treatment can cause positive functional changes; studies have found decreases in overactivation of regions of the limbic system, such as the ACC [183], amygdala [184], and hippocampus [185]. Decreased activity in these regions correlated with improvement in PTSD [186] and social anxiety [184] symptom severity.

The literature reviewed establishes the high prevalence of comorbid SUD-AD and shows the commonality in neuroadaptations present in both conditions. Yet treatments tailored to address this comorbidity have been minimally explored. Further research is therefore needed on treatments for comorbid ADs and SUDs, including studies with a focus on assessing functional changes in the brain. Longitudinal investigations may prove especially useful in investigating treatments, as they would provide information about acute versus long-term changes in these disorders.

Viewing the literature through the RDoC lens highlights several potential routes for improvement in precision treatment of comorbid ADs and SUDs, such as integrated behavioral treatments. Promisingly, a meta-analysis found that integrated AD and SUD treatments outperformed SUD treatments in measures of both substance use and anxiety [187]. Applying prior knowledge of existing therapies to patient populations with AD-SUD comorbidity will help inform development of novel integrated treatments and application of existing behavioral therapies to this population. For example, cognitive behavioral therapy (CBT) has been shown to normalize hyperactivity in fronto-parietal networks, including the PFC, in patients with ADs [188]. Integrated AD-SUD treatments may focus on CBT during the preoccupation/anticipation phase when high anxiety levels exacerbate dysfunction in the PFC and contribute to further drug consumption.

Similarly, medications may have differential efficacies for those with comorbid ADs and SUDs, relative to either condition alone. The RDoC framework predicts that persons with comorbid ADs and alcohol use disorder (AUD) may be more prone to “relief” drinking (to alleviate anxiety) as opposed to “reward” drinking subtypes of AUD. There is some evidence that different medications may have differential efficacy across AUD subtypes; for instance, naltrexone may be more effective for the “reward” drinking subtype [189]. Thus, one could test the possibility of whether medications like acamprosate [190] may be more effective in comorbid ADs and AUD than in AUD alone. This is consistent with evidence that different genetic polymorphisms predict different effects for naltrexone and acamprosate [191] in the treatment of AUD. This type of analysis could be applied to other medication therapies for SUDs, such as for nicotine and cocaine use disorder, that also have evidence of different subtypes elucidated by genetic studies, and for which genetic background modulates brain functional responses in circuits governing disorder-related behaviors [192,193].

One particular area of interest for future research is investigating how biological sex influences comorbid ADs and SUDs, and to what extent differences are genetically determined or influenced by environmental factors. Both men and women with SUDs have a higher risk of mental health problems, but women experience higher comorbid ADs and SUDs than men [194]. This discrepancy is intriguing and warrants further study. The known sex difference in the prevalence of ADs may give insight into which specific SUDs are more likely to be comorbid with ADs. Some types of SUD have widely different prevalence within male vs. female populations. For example, stimulant use disorder is much more prevalent in men [195], while benzodiazepine misuse is more prevalent in women [196]. Further, treatment efficacy may differ across various SUDs in part due to sex differences related to ADs. To study this, one may look at differences in neuroimaging data in men versus women and analyze the findings in parallel with molecular stress signaling, in order to help understand the complex characteristics of comorbid AD-SUD. We noted a relative paucity of studies in this space. There are relative clues from each individual condition; for instance, the amygdala has emerged as a notable region with sex differences in function that have relevance for both trait anxiety [197] and substance use [198].

In addition, investigating the role of sleep in comorbid AD-SUD could provide important insights into the mechanisms driving these conditions. Studies using fMRI and sleep profilers could help clarify the impact of sleep disturbances on brain function and behavior in individuals with both ADs and SUDs. Previous studies have found that sleep apnea is associated with co-occurring anxiety [199,200], with similar findings for insomnia [201]. A recent review found that many types of problematic sleep, including chronic sleep restriction and sleep disorders, can facilitate drug intake and addiction, perhaps via altered interactions between positive and negative valence circuitries [202]. Understanding how sleep disturbances contribute to the development and maintenance of both ADs and SUDs could lead to more integrated treatment approaches that address both sleep and mental health simultaneously.

Finally, the underlying neurobiological basis for the direction of causality between ADs and SUDs could be better understood; it may be particularly important to explore whether the order of onset (“self-medication” of anxiety using substances versus SUD-inducing anxiety disorders) reflects differences in underlying neurobiology. Variables such as the duration of drug use, abstinence periods, relapse rates, and overdose rates should also be included to better understand the progression and outcomes of SUDs. These research aims would help develop more effective treatment and prevention strategies for SUDs and anxiety disorders.

## Data Availability

No new data were created or analyzed in this study. Data sharing is not applicable to this article.
